# The carbon monoxide prodrug oCOm‐21 increases Ca^2+^ sensitivity of the cardiac myofilament

**DOI:** 10.14814/phy2.15974

**Published:** 2024-03-16

**Authors:** Fergus M. Payne, Samantha Nie, Gary M. Diffee, Gerard T. Wilkins, David S. Larsen, Joanne C. Harrison, James C. Baldi, Ivan A. Sammut

**Affiliations:** ^1^ School of Biomedical Sciences, Department of Pharmacology and Toxicology University of Otago Dunedin Otago New Zealand; ^2^ Otago Medical School, Department of Medicine University of Otago Dunedin Otago New Zealand; ^3^ HeartOtago University of Otago Dunedin New Zealand; ^4^ Department of Kinesiology University of Wisconsin‐Madison Madison Wisconsin USA; ^5^ School of Science, Department of Chemistry University of Otago Dunedin Otago New Zealand

**Keywords:** calcium sensitivity, carbon monoxide, heme, myofilament

## Abstract

Patients undergoing cardiopulmonary bypass procedures require inotropic support to improve hemodynamic function and cardiac output. Current inotropes such as dobutamine, can promote arrhythmias, prompting a demand for improved inotropes with little effect on intracellular Ca^2+^ flux. Low‐dose carbon monoxide (CO) induces inotropic effects in perfused hearts. Using the CO‐releasing pro‐drug, oCOm‐21, we investigated if this inotropic effect results from an increase in myofilament Ca^2+^ sensitivity. Male Sprague Dawley rat left ventricular cardiomyocytes were permeabilized, and myofilament force was measured as a function of ‐log [Ca^2+^] (pCa) in the range of 9.0–4.5 under five conditions: vehicle, oCOm‐21, the oCOm‐21 control BP‐21, and levosimendan, (9 cells/group). Ca^2+^ sensitivity was assessed by the Ca^2+^ concentration at which 50% of maximal force is produced (pCa_50_). oCOm‐21, but not BP‐21 significantly increased pCa_50_ compared to vehicle, respectively (pCa_50_ 5.52 vs. 5.47 vs. 5.44; *p* < 0.05). No change in myofilament phosphorylation was seen after oCOm‐21 treatment. Pretreatment of cardiomyocytes with the heme scavenger hemopexin, abolished the Ca^2+^ sensitizing effect of oCOm‐21. These results support the hypothesis that oCOm‐21‐derived CO increases myofilament Ca^2+^ sensitivity through a heme‐dependent mechanism but not by phosphorylation. Further analyses will confirm if this Ca^2+^ sensitizing effect occurs in an intact heart.

## 1 INTRODUCTION

Acute myocardial infarction occurs from an absence of oxygen delivery to the myocardium due to severe coronary artery occlusion and often results in pathological left ventricular (LV) remodeling (Reed et al., 2017). To correct these vascular defects, patients undergo cardiopulmonary bypass procedures, which can impose prolonged ischemic periods resulting in perioperative complications such as stunned myocardium, LV dysfunction and ultimately, heart failure (Kloner et al., 1994; Monaco et al., 2020). A substantial proportion of these cardiac surgical patients will receive inotropes, to reduce the development of low‐output syndromes and sustain adequate organ perfusion (Karami et al., 2020). Inotropes, such as dobutamine or milrinone, increase heart rate and augment the cytosolic Ca^2+^ transient, saturating the Ca^2+^ binding subunit of the myofilament, troponin C (TnC), to increase myofilament cross‐bridge formation (DesJardin & Teerlink, 2021). However, this increase in heart rate and Ca^2+^ flux is associated with a significant increase in myocardial oxygen demand (Bailey et al., 1994; Fellahi et al., 2008; Shabana et al., 2020). Additionally, the enhanced re‐uptake of Ca^2+^ into the sarcoplasmic reticulum may promote store overload induced Ca^2+^ release, resulting in an increased risk of arrhythmias (Said et al., 2008; Zhang & Zhang, 2020).

Myotropes, such as omecamtiv mecarbil and levosimendan, represent a newer class of inotropes that increase LV contractility via direct interaction with the sarcomere to enhance Ca^2+^ sensitivity and actin‐myosin interactions (Edes et al., 1995; Nagy et al., 2015; Psotka et al., 2019). By increasing LV contractility without influencing intracellular Ca^2+^, it has been proposed that these drugs will avoid adverse increases in myocardial oxygen demand and subsequent arrhythmias. However, while the direct myosin activator omecamtiv mecarbil does not alter intracellular Ca^2+^ transients, this myotrope can induce diastolic dysfunction and electrical alternans within therapeutic concentrations (200 μg/kg) (Fülöp et al., 2021). Similarly, the Ca^2+^ sensitizer levosimendan also inhibits phosphodiesterase III, resulting in an increased intracellular Ca^2+^ flux in addition to sensitizing the myofilament to Ca^2+^ (Bokník et al., 1997; Ørstavik et al., 2014).

Carbon monoxide (CO) administered either in gaseous form (<500 ppm) or using low micromolar concentrations of CO‐releasing molecules (CORMs), is a cardioprotective agent that interacts with heme elements to exert vasodilatory, anti‐apoptotic, anti‐inflammatory, and positive inotropic effects (Brouard et al., 2000; Collman et al., 1976; Dugbartey et al., 2021; Otterbein et al., 2000; Sammut et al., 1998). While CORMs have been extensively tested both in vivo and in vitro studies, CORMs such as CORM‐2 and CORM‐3 however, contain a ruthenium transitional metal center which is cytotoxic in cardiomyocyte, renal, and bacterial cell studies (Southam et al., 2018; Winburn et al., 2012), bringing their utility into question. To combat these cytotoxic effects, boron‐ and manganese‐based CORMs were developed (Motterlini et al., 2005) however, these compounds have unpredictable CO release rates and chemical reactivity associated with the parent compound (Bauer, Yang, et al., 2023; Bauer, Yuan, et al., 2023). A new organic CO‐releasing prodrug, oCOm‐21, has been shown to rapidly release CO at physiological conditions (*t*_1/2_ = 19 min; pH 7.4) and is non‐cytotoxic at concentrations <20 μM (Kueh et al., 2017; Kueh et al., 2020). Low concentrations of oCOm‐21 (1–10 μM) increase vasodilation in isolated aortic rings and LV systolic pressure in a Langendorff‐perfused rat heart model (Thwaite et al., 2021; Kueh et al., 2017) with no evidence of arrhythmia formation or a change in heart rate. While the positive inotropic mechanism of oCOm‐21 is still uncertain, the absence of arrhythmogenicity and chronotropic effect suggests that oCOm‐21 may be directly interacting with the myofilament.

Given the evident binding affinity of CO for heme elements within various hemoproteins (Collman et al., 1976), we hypothesized that oCOm‐21 increases Ca^2+^ sensitivity via heme interactions. The myotropic effects of oCOm‐21 in a permeabilized cardiomyocyte preparation were compared to the established Ca^2+^ sensitizer levosimendan. To determine if oCOm‐21 derived‐CO directly interacts with the myofilament, this study tested the Ca^2+^ sensitivity of permeabilized cardiomyocytes in the presence of oCOm‐21 (3 and 10 μM) and the spent CO by‐product (BP‐21). Potential mechanisms behind alterations in Ca^2+^ sensitivity were also investigated. Sex as a biological variable was considered in this myofilament study, however prior work has shown no sex differences in either the baseline LV contractile function in isolated hearts, or in myofilament protein phosphorylation, and Ca^2+^ sensitivity of permeabilized myofilaments of mice (≤12 months of age) (Kane et al., 2020).

## 2 MATERIALS AND METHODS

### 2.1 Materials

oCOm‐21 and BP‐21 (CO‐depleted oCOm‐21 byproduct), were synthesized by the Larsen group, Department of Chemistry, University of Otago (Kueh et al., 2017). Levosimendan (L5545) and hemopexin (H9291), phenylmethylsulfonyl fluoride (78830), Protease Inhibitor Cocktail (P8340), Phosphatase Inhibitor Cocktail (P5726), oxalic acid, and Spurr's epoxy resin were purchased from Sigma‐Aldrich. HALT protease and phosphatase inhibitor cocktail 100× (78430), Triton X‐100 (28314), Pro‐Q™ Diamond Phosphoprotein gel stain (P33300), SYPRO® Ruby Protein Gel Stain (S12000), and PeppermintStick™ Phosphoprotein molecular weight Standard (P27167) were purchased from Thermo Fisher Scientific.

### 2.2 Animal ethics and care

Male Sprague Dawley rats (320–360 g, *n* = 9 animals) were obtained from the University of Otago's Biological Research Facility. All procedures described were carried out under institutional approval granted (AUP 21–63) in accordance with the “Guidelines on the Care and Use of Laboratory Animals” set out by the University of Otago Animal Ethics Committee. Hearts were excised under deep anesthesia using 5% isoflurane, and the LV immediately separated and frozen in liquid nitrogen.

### 2.3 Cardiomyocyte force‐pCa measurements

**FIGURE 1:**
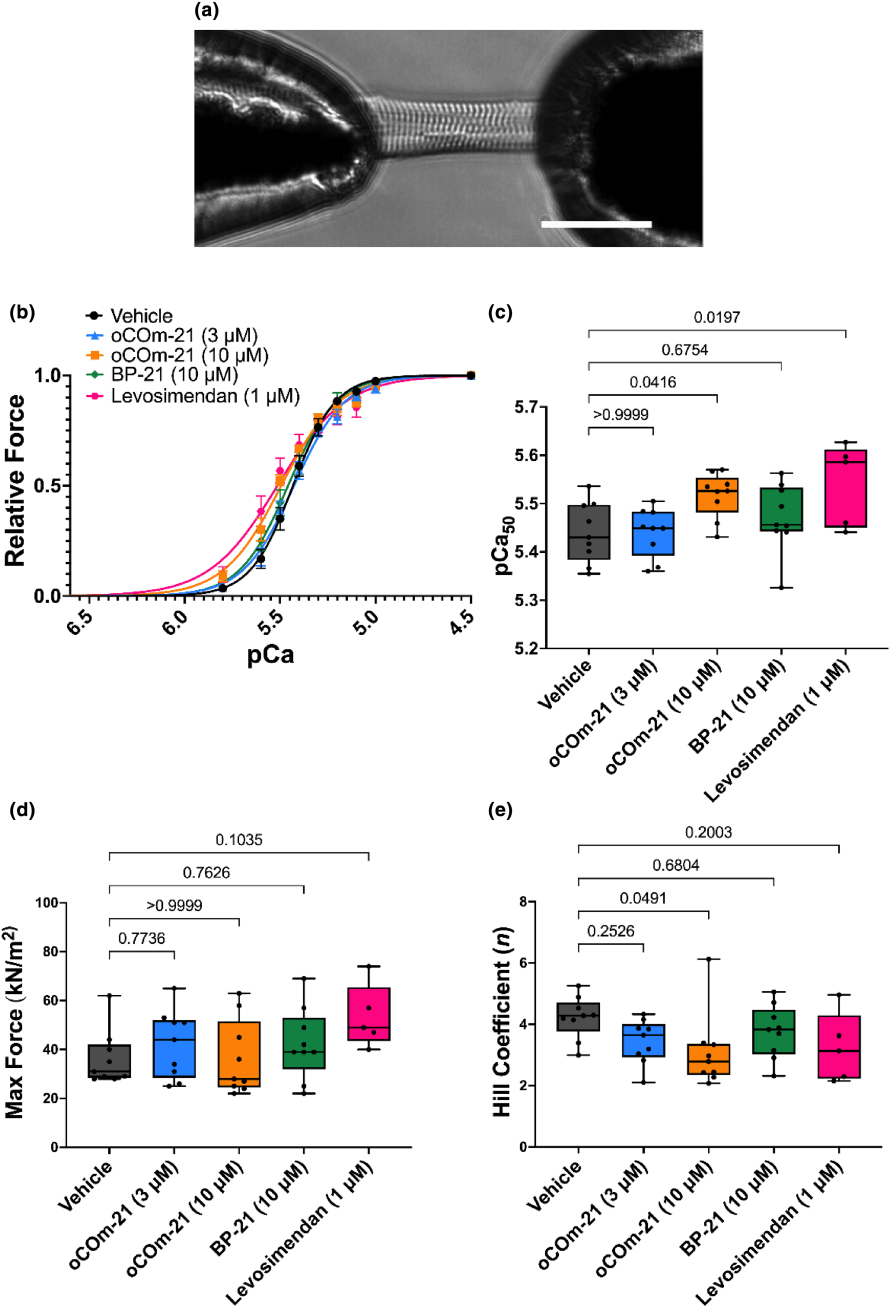
oCOm‐21 (10 μM) increases myofilament Ca^2+^ sensitivity. (a) Example of a permeabilized cardiomyocyte, glued to two micro‐pins connected to a force transducer and piezoelectric motor arm at 20× magnification. Scale bar shown represents 50 μm. (b) Force‐pCa relationships of permeabilized cardiomyocytes treated with either vehicle, oCOm‐21 (3, 10 μM), BP‐21 (10 μM), or levosimendan (1 μM). (c) pCa_50_ values for each treatment group. (d) Maximal active force values for each treatment group. (e) Hill coefficient (*n*) values for each treatment group. Individual cell data points in (c–e) are denoted using black solid circles. Data in 1B is expressed as mean ± S.E.M while data in (c–e) are expressed as Box and Whiskers plots representing the median and interquartile range, Experimental repeat *n* = 5–9 cells/group obtained from three individual hearts. Significance was determined using a One‐way ANOVA with Dunnett post hoc test and *p* is represented on each experimental group.

Permeabilized LV cardiomyocytes isolated from male Sprague Dawley rats were used to examine changes in Ca^2+^ sensitivity as described previously (Greenman et al., 2022; Ng et al., 2022). Briefly, frozen LV tissue was homogenized in a Relax buffer (100 mM KCl, 1.75 mM EGTA, 10 mM imidazole, 4 mM ATP, 5 mM MgCl_2_, pH 7) with HALT protease and phosphatase inhibitor cocktail. The myocyte suspension was subsequently treated with 1% Triton X‐100 for 8 min to yield a pool of permeabilized cardiomyocytes. Permeabilized cardiomyocytes were then washed twice with Relax buffer to remove the detergent and cytosolic/membrane‐bound components and stored on ice. Individual permeabilized cardiomyocytes were mounted to an inverted phase contrast microscope (Nikon Eclipse Ts2R, Coherent, Australia) and adhered to 100 μm pins connected to a force transducer and a piezoelectric motor arm (Aurora Scientific, 406a model, Aurora, ON, Canada) using silicone glue within a micro‐organ bath containing relax buffer at 15°C. Each cardiomyocyte preparation was adjusted to a sarcomere length of 2.2 μm using micromanipulators while the cells were immersed in a well containing pCa 9.0 (7 mM EGTA, 20 mM imidazole, 5.42 mM MgCl_2_, 79.16 mM KCl, 10^−9^ M free Ca^2+^, 14.5 mM creatine phosphate, 4.74 mM ATP, pH 7). Cell width, length, and sarcomere length were monitored throughout the experiment using an IDS camera and *VSL 900B* software (Aurora Scientific) (Figure 1a).

Cardiomyocytes were immersed in a randomly‐ordered series of submaximal pCa solutions (pCa 5.8–5.0) and allowed to generate steady state force until a plateau was established. The cardiomyocytes were then slackened by 20% of their initial length to measure force production before being transferred back to the relaxing pCa 9.0 solution to allow myofilament relaxation. Force was measured as a function of pCa (−log [Ca^2+^]) in the range of pCa 9.0–4.5 under 5 test conditions: vehicle control, oCOm‐21 at 3 or 10 μM, the CO‐depleted control BP‐21 (10 μM), and levosimendan (1 μM). The force generated in pCa 4.5 (7 mM EGTA, 20 mM imidazole, 5.26 mM MgCl_2_, 64 mM KCl, 10^−4.5^ M free Ca^2+^, 14.5 mM creatine phosphate, 4.81 mM ATP, pH 7) was used to assess maximal force production. This was followed by eight, random selected submaximal pCa solutions (pCa 5.8–5.0), with a pCa 4.5 measurement conducted after every fourth measurement to assess the performance of the cardiomyocyte. The Ca^2+^ concentration at which 50% of maximal force is produced (pCa_50_), was used to measure Ca^2+^ sensitivity. Cardiomyocytes which declined in force by 15% from the beginning of the experiment were discarded. In a separate set of experiments, permeabilized cardiomyocytes were incubated with the heme scavenger hemopexin (HPX) (1 μM) in pCa 9.0. HPX‐treated cells were then incubated with oCOm‐21 (10 μM) to determine whether heme was required for the observed Ca^2+^ sensitizing effect.

Maximal active force was normalized to the cross‐sectional area of the cell based on a circular structure (Cross‐sectional area = 3.14 × (1/2cell width)^2^). Force generation at each pCa was expressed as a fraction of the maximal force obtained for that cell under the same conditions and data was analyzed using the Hill equation:

$$\mathrm{Log}\left[p_{\mathrm{rel}}/\left(1-P_{\mathrm{rel}}\right)\right]=n\left(\log\left[{\mathrm{Ca}}^{2+}\right]+k\right)$$

*P*_rel_ is force expressed as a fraction of maximal force, *n* is the Hill coefficient, and *k* is the intercept of the fitted line with the x‐axis corresponding to the pCa_50_ (Hofmann et al., 1991). Using constants derived from the Hill equation, force data was fitted using Prism software (GraphPad Software Inc., La Jolla, CA, USA) with the following equation (Diffee et al., 2001):

$$P_{\mathrm{rel}}={\left[{\mathrm{Ca}}^{2+}\right]}^{n}/\left(k^{n}+{\left[{\mathrm{Ca}}^{2+}\right]}^{n}\right)$$

### 2.4 Pro‐Q™ diamond phosphoprotein gel staining

Pro‐Q™ Diamond Phosphoprotein gel stain was used to determine the effect of oCOm‐21 on myofilament protein phosphorylation in the permeabilized cardiomyocyte. Permeabilized cardiomyocytes were homogenized in RIPA buffer (50 mM NaCl, 1% SDS, 1% Triton X‐100, 1 mM edetate disodium, pH 7.4 with HCl) containing phenylmethylsulfonyl fluoride, protease inhibitor cocktail and phosphatase inhibitors. Permeabilized cardiomyocytes were treated with either vehicle or oCOm‐21 (3, 10 μM) for 20 minutes and protein samples were then prepared for gel electrophoresis and denatured at 70°C for 10 minutes. Protein concentrations were determined by a Lowry assay (Sammut et al., 2001) and 20 μg of protein was loaded into each well onto a gradient (4%–20%) gel (Mini‐PROTEAN® TGX™ Precast Gels, Bio‐Rad # 4561096) and run with 25 mM Tris Base, 192 mM glycine, 1% SDS buffer. PeppermintStick™ Phosphoprotein Molecular Weight Standard (Invitrogen) was also added on neighboring lanes to act as both a molecule weight marker and a positive control. Electrophoresis was performed at 80 V for an initial 5 min, followed by an increase to 120 V (1 h). After gel electrophoresis, gels were fixed overnight in 50% methanol and 10% acetic acid to remove SDS. Gels were then washed to remove the fixing solution and subsequently stained with Pro‐Q™ Diamond gel stain. Gels were protected from light and stained for 60 min. Gels were de‐stained using the Pro‐Q™ Diamond Phosphoprotein Gel Destaining Solution for 90 min to reduce background staining. Gels were then imaged using an Amersham ImageQuant 800 (Cytiva, Auckland, NZ) at 535 nm with a 0.2 s exposure.

To quantify total protein, gels were stained with SYPRO® Ruby Protein Gel Stain overnight and subsequently washed with 10% and 7% acetic acid before imaging. SYPRO® Ruby‐stained gels were imaged using the Amersham ImageQuant 800 at 460 nm with a 0.2 s exposure.

Protein band intensities were quantified using Image Lab Software (Bio‐Rad v.6.1). Phosphorylation was normalized to total protein using the ratio of the signal intensity of the phospho‐stained band divided by the SYPRO®‐stained band.

### 2.5 Microscopy

To investigate the presence of mitochondria, permeabilized cardiomyocytes were prepared for transmission electron microscopy (Sammut et al., 2001). Briefly, permeabilized cardiomyocytes were incubated in relax buffer with 2.5% glutaraldehyde for 2 h at 4°C and for a further 30 min at room temperature before being pelleted at 15,000 *g* for 2 min. The pellet was resuspended in Relax buffer and washed before pelleting at 5000 *g* and embedded in 3% low gelling‐temperature agarose at 4°C for 10 min. The pellet was then immersed in 1% osmium tetroxide in relax buffer for 1.5 h and subsequently dehydrated through an ethanol series (50%–100%) and transferred to propylene oxide. The pellet was then immersed in Spurr's epoxy resin and allowed to cure for 24 h at 60°C before being sectioned. Ultra‐thin sections were stained with uranyl acetate and lead citrate and examined using a Phillips CM100 TEM.

### 2.6 Cardiomyocyte heme content

Heme content in permeabilized LV cardiomyocytes was determined by an oxalic acid based fluorescent measurement of the heme precursor protoporphyrin IX (PPIX). Samples were either pre‐treated with HPX (1 μM) or vehicle (0.15 M NaCl in phosphate buffered solution). Briefly, sample protein content was determined using the Lowry assay as previously described (Sammut et al., 2001). Oxalic acid (2 M) was added to 10 μg of protein and heated to 100°C for 30 min to generate fluorescent PPIX from heme. Protein samples were subsequently centrifuged at 3630 *g* for 5 min at 4°C and added to a 96‐well plate. Fluorescence excitation and emission were set at 405 and 631 nm, respectively, and measured using the i3X SpectraMax (Molecular Devices, San Jose, USA). Background fluorescence was evaluated in parallel in non‐boiled protein samples.

### 2.7 Statistical analysis

The researchers conducting these experiments were blinded to the identity of the experimental agent until all data had been analyzed and graphed.

Results are expressed as means ± S.E.M and were analyzed either using an unpaired *t*‐test or a one‐way ANOVA with a Dunnett or Bonferroni post hoc as appropriate to determine significance between groups where *p* < 0.05 was considered statistically significant. All statistical analysis were performed using GraphPad Prism v.9 (GraphPad Software, La Jolla, CA).

## 3 RESULTS

### 3.1 oCOm‐21 increases Ca^2+^ sensitivity of the myofilament

Ca^2+^ sensitivity was assessed in permeabilized cardiomyocytes treated with either vehicle, oCOm‐21 (3, 10 μM), BP‐21 (10 μM) or levosimendan (1 μM). Incubation with oCOm‐21 at 10 μM, increased pCa_50_ compared to vehicle (5.52 vs. 5.44 respectively; *p* < 0.05) while reducing (*p* < 0.05) the Hill Coefficient (*n*), (Figure 1b,c,e) while 3 μM oCOm‐21 did not alter pCa_50_ (5.44 vs. 5.44; *p* > 0.05) (Figure 1b,c). BP‐21 did not increase pCa_50_ compared to vehicle (5.47 vs. 5.44 respectively; *p* > 0.05). Comparably, levosimendan increased pCa_50_ compared to vehicle (5.44 vs. 5.54 respectively; *p* < 0.05). No other treatment groups increased maximal active force of the cardiomyocyte or altered co‐operativity of Ca^2+^ binding compared to vehicle (*p* > 0.05) (Figure 1d,e).

### 3.2 oCOm‐21 does not impact the phosphorylation status of the myofilament

**FIGURE 2:**
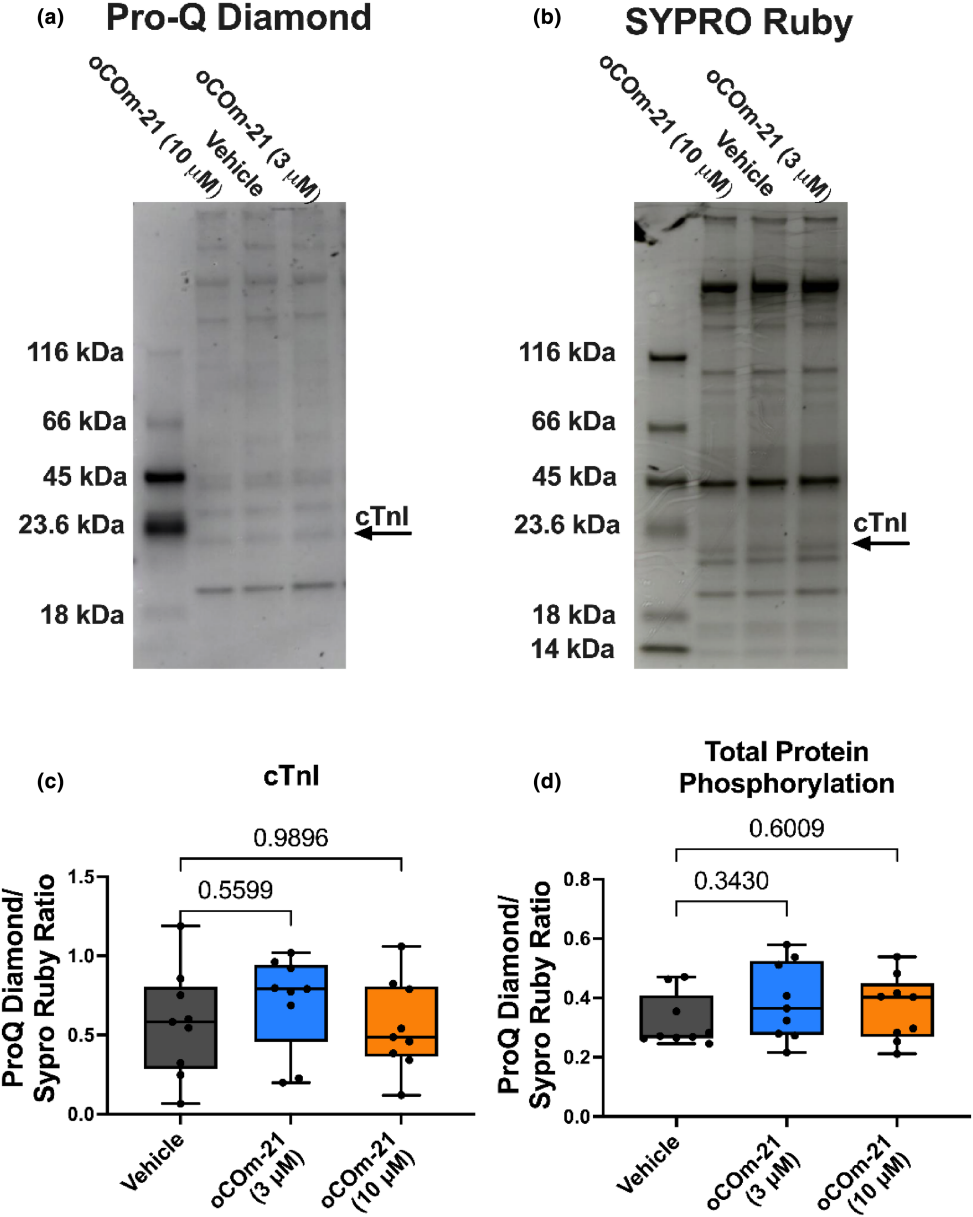
oCOm‐21 does not alter myofilament phosphorylation in a permeabilized cardiomyocyte. (a) Representative Pro‐Q™ Diamond protein phosphorylation‐stained gradient gel and, (b) subsequent SYPRO® Ruby total protein staining. (c) Ratio of Pro‐Q™ Diamond and SYPRO® Ruby signal for cTnI. (d) Ratio of Pro‐Q™ Diamond and SYPRO® Ruby signal for total protein phosphorylation. Individual data points in (c, d) are denoted using black solid circles. Data are expressed as a Box and Whiskers plot representing the median and interquartile range, *n* = 9 hearts/group. Significance was determined using a One‐way ANOVA with Dunnett post hoc test and *p* is represented on each experimental group.

In order to assess a potential mechanism by which oCOm‐21 alters Ca^2+^ sensitivity, the total phosphorylation status and specifically, the phosphorylation of cardiac troponin I (cTnI) of permeabilized cardiomyocytes were assessed. No change in total protein phosphorylation or cTnI phosphorylation was observed with oCOm‐21 at either concentration compared to vehicle (*p* > 0.05) (Figure 2a–d).

### 3.3 Hemopexin abolishes the Ca^2+^ sensitizing effect of oCOm‐21

**FIGURE 3:**
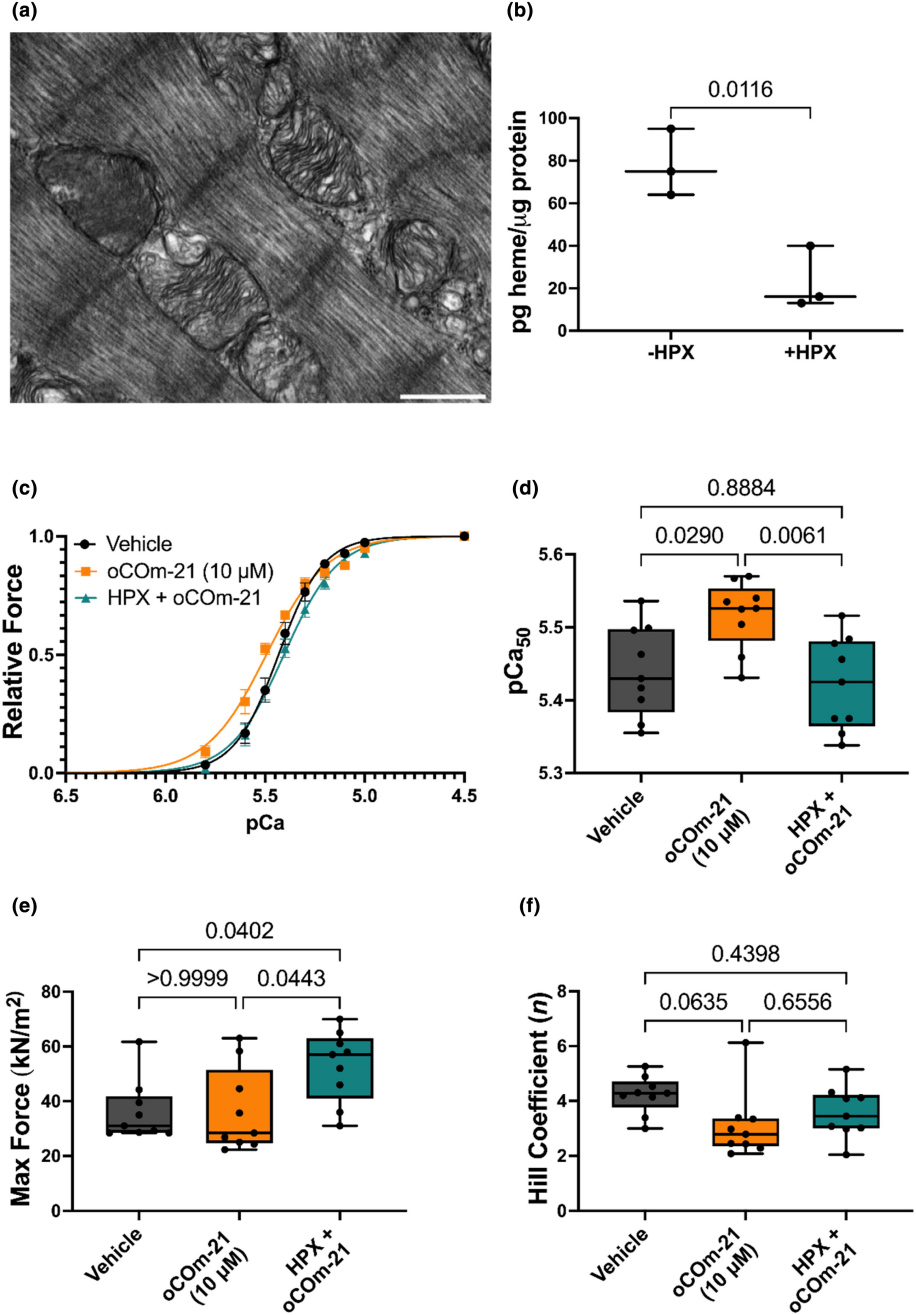
HPX prevents the Ca^2+^ sensitizing effect of oCOm‐21 (10 μM). (a) Electron micrograph of interfibrillar mitochondrial structures in a permeabilized cardiomyocyte at 33,000× magnification; scale bar shown represents 500 nm. (b) Fluorescence analysis of heme content in both untreated and HPX treated (1 μM) permeabilized cardiomyocytes. (c) Force‐pCa relationships of permeabilized cardiomyocytes treated with vehicle, oCOm‐21 (10 μM) or HPX (1 μM) + oCOm‐21 (10 μM). (d) pCa_50_ values for each treatment. (e) Max force values for each treatment. (f) Hill coefficient values for each treatment group. Individual cell data points in (d–f) are denoted using black solid circles. Data in (b) is expressed as a Box and Whiskers plot representing the median and interquartile range, *n* = 3 hearts/group, significance determined by an unpaired *t*‐test. Data in (c) is expressed as the means ± S.E.M while data in (d–f) is expressed as a Box and Whiskers plot representing the median and interquartile range, *n* = 9 cells/group obtained from three hearts. Significance was determined using a One‐way ANOVA with Bonferroni post hoc test and *p* is represented on each experimental group.

Electron microscopical studies confirmed the presence of mitochondrial remnants throughout the myofilament (Figure 3a), providing evidence for an intracellular store of free heme within the permeabilized cardiomyocyte. To confirm this ultrastructural finding, heme content was quantified in permeabilized cardiomyocytes fluorometrically. Heme content was significantly reduced to the lowest limit of detection with the heme scavenger HPX, indicating free heme exists within these permeabilized cells (Figure 3b). Removal of free heme, the binding target for CO, using HPX before incubation with oCOm‐21 prevented the Ca^2+^ sensitizing effect and dropped pCa_50_ (5.52–5.42; *p* < 0.01) indicating a heme‐dependent mechanism of oCOm‐21 (Figure 3b,c). Additionally, HPX pretreatment increased maximal force of the cardiomyocyte compared to vehicle and oCOm‐21 (*p* < 0.05) (Figure 3d–f).

## 4 DISCUSSION

This study investigated the Ca^2+^ sensitizing effects of a CO‐releasing prodrug, oCOm‐21. The addition of oCOm‐21 to a permeabilized cardiomyocyte preparation increased pCa_50_ to the same degree as the clinically approved drug levosimendan, an established Ca^2+^ sensitizer. Importantly BP‐21, the CO‐depleted by‐product of oCOm‐21, did not alter pCa_50_. Moreover, when CO binding targets were removed with the free‐heme scavenger HPX, the Ca^2+^ sensitizing effects of oCOm‐21 were abolished. These findings support our hypothesis that oCOm‐21 increases Ca^2+^ sensitivity through a CO‐heme‐dependent mechanism.

Our group (Greenman et al., 2021; Ng et al., 2022) and others (Budde et al., 2021; Sevrieva et al., 2023; van der Velden et al., 2003) have shown that phosphorylation of myofilament proteins (including cTnI and myosin binding protein C) is associated with changes in Ca^2+^ sensitivity. CO activates the soluble guanylate cyclase (sGC) pathway to phosphorylate cytosolic (e.g., L‐type Ca^2+^ channel, ryanodine receptor) and myofilament proteins such as TnI at Ser23/24 (Layland et al., 2002; Stone & Marletta, 1994). Inhibition of sGC activity with (1H‐[1,2,4]oxadiazolo‐[4,3‐a]quinoxaline‐1‐one) (ODQ) in perfused rat hearts inhibited the inotropic effect of the earlier CO prodrug CORM‐3 (Musameh et al., 2006), suggesting that CO elicited an inotropic effect via transient phosphorylation of cytosolic and myofilament proteins. However, the present study showed that oCOm‐21 increased pCa_50_ in permeabilized cardiomyocyte preparations, where the cytosolic enzyme sGC should not be present (Gao et al., 2012; Solaro et al., 1971; van der Velden et al., 2003). Indeed, Pro‐Q diamond staining showed that oCOm‐21 did not change the phosphorylation status of the myofilament proteome in permeabilized cell homogenates. These discrepancies may indicate that oCOm‐21 induces protein phosphorylation affecting contractility and myofilament Ca^2+^ sensitivity within *intact* preparations. The novel finding of this study is that oCOm‐21 increased Ca^2+^ sensitivity without affecting phosphorylation of the myofilament.

Direct binding of CO to myofilament proteins, as seen with other myotropes including TA1 (He et al., 2022), levosimendan (Edes et al., 1995; Haikala et al., 1995) and EMD 57033 (Li et al., 2000) may explain the increased pCa_50_ identified with oCOm‐21. The present study did not confirm a direct binding mechanism of oCOm‐21, however the established heme dependency for the Ca^2+^ sensitization effect may provide insight. Leclerc et al. (1993), identified a direct interaction of the CO‐heme complex with skeletal TnC and other Ca^2+^ binding proteins. It is possible that CO derived from oCOm‐21 binds heme and interacts with TnC to increase Ca^2+^ sensitivity. Permeabilization of cardiomyocytes, removes cytosolic, and membrane‐bound components (Solaro et al., 1971), however, it was unclear whether residual fragments of interfibrillar mitochondria which contain heme (Lange et al., 1999) were present. Therefore, transmission electron microscopy was used to determine whether the permeabilized cardiomyocytes in these preparations retained mitochondria. Our micrographs visually confirmed the presence of mitochondrial components in the permeabilized cardiomyocyte preparations, as established sources of free heme for CO interactions (Barth et al., 1992; Medlock et al., 2015; Swenson et al., 2020). Heme content in permeabilized cardiomyocytes was reduced upon treatment with HPX, further indicating pools of free heme for CO interactions are retained following cardiomyocyte permeabilization (Detzel et al., 2021). Moreover, HPX abolished the Ca^2+^ sensitizing effect of oCOm‐21, demonstrating that the elimination of free heme from these myofilament preparations, nullifies the effect of oCOm‐21 on myofilament contractility. These findings suggest that CO interacts with mitochondrial‐derived heme stores in a permeabilized cardiomyocyte preparation to increase pCa_50_. It is however impossible to speculate whether heme is derived from the mitochondrial hemoprotein degeneration or through heme generation by mitochondrial remnants.

We were surprised to find that HPX also increased the maximal active force of the cardiomyocyte. Maximal active force produced is determined by a combination of the maximal number of actin‐myosin cross bridges formed and the rigidity of the cross‐bridges (Linari et al., 2007). Alvarado et al. ( 2015) showed that the addition of heme to myofilaments reduced maximal active force of cardiomyocytes due to oxidation on myosin light chain 1 which could be reversed upon HPX treatment. In this context, the addition of HPX in our experiments may have removed basal concentrations of heme from the myofilament thereby improving maximal active force.

Interestingly, oCOm‐21 reduced myofilament co‐operativity as identified by the decreased Hill slope. Co‐operativity for thin filament activation is described as the result of a specific event (e.g., Ca^2+^ binding to TnC), nonlinearly favoring the occurrence of that same event (Razumova et al., 2000). In other words, as more Ca^2+^ binds to TnC, the affinity of neighboring TnC proteins for Ca^2+^ increases, therefore, force production is dependent on co‐operativity (Farman et al., 2010). Ca^2+^ sensitizers such as levosimendan have no impact on cooperativity (Szilágyi et al., 2004; Van Hees et al., 2011) however, omecamtiv mecarbil increases Ca^2+^ sensitivity while decreasing cooperativity (Nagy et al., 2015), comparable to oCOm‐21. Both omecamtiv mecarbil and oCOm‐21 increase force production at [Ca^2+^] below the pCa_50_ but produce similar force at [Ca^2+^] above the pCa_50_, resulting in the ascending portion of the force‐pCa relationship becoming less steep, reducing co‐operativity. The observed increase in force production at lower [Ca^2+^] may prolong contractions, consequently impairing diastolic function. Therefore, further studies should investigate the impact of oCOm‐21 on diastolic function and if the Ca^2+^ sensitizing effect still occurs at lower [Ca^2+^] in an intact heart preparation.

## 5 LIMITATIONS

A limitation to this study was that only permeabilized cardiomyocyte preparations were utilized to examine the Ca^2+^ sensitizing effects of oCOm‐21. CO is highly pleiotropic and interacts with a variety of heme‐containing proteins such as hemoglobin, myoglobin, and mitochondrial complexes (Coletta et al., 1985; Dugbartey et al., 2021). By using a permeabilized preparation, these components were removed (Solaro et al., 1971), suggesting that CO interacts with the myofilament. The micrographical evidence provided (Figure 3) however showed that mitochondrial elements were still present in these cells and that heme was implicated in this CO response. It is unclear however, from the use of this permeabilised cell model whether CO is still able to preferentially interact with the myofilament over other hemoproteins within an intact cell. While heme levels could be quantified in permeabilized cells, incubation with HPX, reduced heme concentrations to the lowest limit of detection making it difficult to accurately determine concentrations of free heme in the HPX treated cardiomyocytes. This finding however, confirms that HPX treatment, significantly reduced the availability of free heme in the subsequent Force‐pCa experiments provided in Figure 3.

Consideration was also given to the inclusion of myofilaments isolated from female hearts in this study. Similar work conducted by Howlett's group in a mouse model however, found no difference in Ca^2+^ sensitivity or myofilament protein phosphorylation as a consequence of sex (Kane et al., 2020). This lack of dimorphism in myofilament contractility is supported by earlier work conducted in myofilaments isolated from a cat cardiac model (Petre et al., 2007) and in wild type exercise‐trained or sedentary mice (Najafi et al., 2015). Schuldt et al. (2021) also recently found that tubulin was the only sarcomeric protein whose expression was affected by sex in cardiomyocytes harvested from patients receiving LV outflow tract obstruction reduction surgeries.

In summary, these results indicate that in the presence of heme, myofilament Ca^2+^ sensitivity is increased when oCOm‐21 is applied directly to a permeabilized cardiomyocyte preparation. This suggests that liberated CO interacts with myofilament proteins through a heme‐dependent mechanism to elicit this effect rather than through a posttranslational modification. This interaction will be examined using mass‐spectrophotometric analysis. Further investigations will also establish whether this Ca^2+^ sensitizing effect is conserved in treated hearts and if oCOm‐21 alters Ca^2+^ cycling. These findings add to our understanding of the clinically valuable effects of low dose CO administration. While high concentrations of CO are clearly cardiotoxic, this study suggests that low concentrations of the oCOms have positive valuable myotropic effects which may be used to safely improve myocardial contractility in cardiac injury.

## AUTHOR CONTRIBUTIONS

Fergus M. Payne, Gary M. Diffee, James C. Baldi, and Ivan A. Sammut designed the experiments. Fergus M. Payne performed the experiments for Figures 1 and 3 and wrote the manuscript draft. Samantha Nie performed the experiments for Figure 2. Fergus M. Payne, Samantha Nie and James C. Baldi analyzed and interpreted data. Joanne C. Harrison and Ivan A. Sammut, ensured that the test conditions were blinded. David S. Larsen synthesized oCOm‐21 and BP‐21 in collaboration with Joanne C. Harrison and Ivan A. Sammut. All authors reviewed and edited the manuscript and approved the final version of the manuscript.

## FUNDING INFORMATION

This work was supported by a University of Otago Research Grant, Grant/Award Number: ORG0122‐0323 (to I.A.S, J.C.B. and J.C.H.) and a Health Research Council of New Zealand Grant, Grant/Award No: 20/274 (to I.A.S, J.C.H, G.T.W. and D.S.L.).

## CONFLICT OF INTEREST STATEMENT

The authors declare no competing financial interests.

## Data Availability

All data are presented in Figures 1, 2, 3 but are also available upon request.
